# Supplementary material for: Label-free multimodal optical biopsy reveals biomolecular and morphological features of diabetic kidney tissue in 2D and 3D
Source: Nat Commun. 2025 May 15;16:4509. doi: 10.1038/s41467-025-59163-w (PMC12081717; doi:10.1038/s41467-025-59163-w)
Supplement: Supplementary file 2 — Reporting Summary [file 41467_2025_59163_MOESM2_ESM.pdf]

nature portfolio

Corresponding author(s): Lingyan Shi

Last updated by author(s): Mar 20, 2025

Reporting Summary

Nature Portfolio wishes to improve the reproducibility of the work that we publish. This form provides structure for consistency and transparency in reporting. For further information on Nature Portfolio policies, see our Editorial Policies and the Editorial Policy Checklist.

Statistics

For all statistical analyses, confirm that the following items are present in the figure legend, table legend, main text, or Methods section.

n/a	Confirmed	
☐	☒	The exact sample size (*n*) for each experimental group/condition, given as a discrete number and unit of measurement
☐	☒	A statement on whether measurements were taken from distinct samples or whether the same sample was measured repeatedly
☐	☒	The statistical test(s) used AND whether they are one- or two-sided<br>*Only common tests should be described solely by name; describe more complex techniques in the Methods section.*
☐	☒	A description of all covariates tested
☐	☒	A description of any assumptions or corrections, such as tests of normality and adjustment for multiple comparisons
☐	☒	A full description of the statistical parameters including central tendency (e.g. means) or other basic estimates (e.g. regression coefficient) AND variation (e.g. standard deviation) or associated estimates of uncertainty (e.g. confidence intervals)
☐	☒	For null hypothesis testing, the test statistic (e.g. *F*, *t*, *r*) with confidence intervals, effect sizes, degrees of freedom and *P* value noted<br>*Give P values as exact values whenever suitable.*
☒	☐	For Bayesian analysis, information on the choice of priors and Markov chain Monte Carlo settings
☒	☐	For hierarchical and complex designs, identification of the appropriate level for tests and full reporting of outcomes
☒	☐	Estimates of effect sizes (e.g. Cohen's *d*, Pearson's *r*), indicating how they were calculated

Our web collection on statistics for biologists contains articles on many of the points above.

Software and code

Policy information about availability of computer code

Data collection	Imaging data were collected using Fluoview FV31S-SW by Olympus
Data analysis	Commercial software involved in data analysis are HALO AI (by IndicaLabs), MATLAB R2023b (by Mathworks), and FIJI. Custom code was written in MATLAB R2023b

For manuscripts utilizing custom algorithms or software that are central to the research but not yet described in published literature, software must be made available to editors and reviewers. We strongly encourage code deposition in a community repository (e.g. GitHub). See the Nature Portfolio guidelines for submitting code & software for further information.

Data

Policy information about availability of data

All manuscripts must include a data availability statement. This statement should provide the following information, where applicable:

- Accession codes, unique identifiers, or web links for publicly available datasets
- A description of any restrictions on data availability
- For clinical datasets or third party data, please ensure that the statement adheres to our policy

A source data file for plotted data is supplied in the supplementary materials as an excel spreadsheet. Processed images of deidentified human samples can be accessed from code examples on the Shi lab github (https://github.com/lingyanshi2020).

Research involving human participants, their data, or biological material

Policy information about studies with human participants or human data. See also policy information about sex, gender (identity/presentation), and sexual orientation and race, ethnicity and racism.

Reporting on sex and gender	sex and gender were not considered in this study due to limited sample availability, pervasiveness of diabetic kidney disease epidemiology and low diagnositic efficiency (meaning that although diabetic kidney symptoms may appear at any time, it may go undiagnosed for extended periods). Additionally, when selecting the samples, this data was occasionally not available.
Reporting on race, ethnicity, or other socially relevant groupings	Race, ethnicity, and other social groupings were not considered in this study. Additionally, when selecting the samples, this data was occasionally not available.
Population characteristics	No covariate criteria were considered due to limited sample availability, pervasiveness of diabetic kidney disease epidemiology and low diagnositic efficiency
Recruitment	Samples were retrieved from archived tissue banks with the primary categorization of diabetic versus non-diabetic diagnosis.
Ethics oversight	Washington University School of Medicine in St. Louis under an approved IRB protocol (IRB 201102312).

Note that full information on the approval of the study protocol must also be provided in the manuscript.

Field-specific reporting

Please select the one below that is the best fit for your research. If you are not sure, read the appropriate sections before making your selection.

- [x] Life sciences
- [ ] Behavioural & social sciences
- [ ] Ecological, evolutionary & environmental sciences

For a reference copy of the document with all sections, see nature.com/documents/nr-reporting-summary-flat.pdf

Life sciences study design

All studies must disclose on these points even when the disclosure is negative.

Sample size	various regions of interest (e.g. glomeruli) were derived from biological replicates (various slide slices from the same biopsy/necropsy) originating from 3 diabetic subjects and 3 non-diabetic (control) subjects. These specific numbers are displayed in the appropriate sections.
Data exclusions	some regions of interest were excluded due to physical constraints (e.g. deformations in the sample due to sectioning or other processing, or technical difficulties arising from laser microscope operation such as excessive noise or poor signal quality)
Replication	The objective of this study is to demonstrate the technical ability of the label-free platform in analyzing structural/spatial and biomolecular data. Samples were fully utilized and no hold-out or saved portions of the datasets were reserved for back-testing. Due to limited sample availability and quality, further replication studies to validate biomarkers are warranted provided future funding and interest.
Randomization	Samples were not randomized. The diagnosis of diabetic vs non-diabetic was made apparent beforehand, and was in fact necessary in order to select the samples for this study. For comparative analyses, data from all regions of interest were pooled.
Blinding	The diagnosis of diabetic vs non-diabetic was made apparent beforehand, and was in fact necessary in order to select the samples for this study. Additionally, control samples were derived via necropsy from patients with no prior diagnosis of diabetic kidney disease or diabetic nephropathy, while diabetic samples were derived via needle core biopsy from living patients. Therefore the samples being imaged had stark size differences (needle core biopsies are long and thin while necropsy block are relatively large)

Reporting for specific materials, systems and methods

We require information from authors about some types of materials, experimental systems and methods used in many studies. Here, indicate whether each material, system or method listed is relevant to your study. If you are not sure if a list item applies to your research, read the appropriate section before selecting a response.

Materials & experimental systems

n/a	Involved in the study
☒	☐ Antibodies
☒	☐ Eukaryotic cell lines
☒	☐ Palaeontology and archaeology
☒	☐ Animals and other organisms
☒	☐ Clinical data
☒	☐ Dual use research of concern
☒	☐ Plants

Methods

n/a	Involved in the study
☒	☐ ChIP-seq
☒	☐ Flow cytometry
☒	☐ MRI-based neuroimaging

Plants

Seed stocks	*Report on the source of all seed stocks or other plant material used. If applicable, state the seed stock centre and catalogue number. If plant specimens were collected from the field, describe the collection location, date and sampling procedures.*
Novel plant genotypes	*Describe the methods by which all novel plant genotypes were produced. This includes those generated by transgenic approaches, gene editing, chemical/radiation-based mutagenesis and hybridization. For transgenic lines, describe the transformation method, the number of independent lines analyzed and the generation upon which experiments were performed. For gene-edited lines, describe the editor used, the endogenous sequence targeted for editing, the targeting guide RNA sequence (if applicable) and how the editor was applied.*
Authentication	*Describe any authentication procedures for each seed stock used or novel genotype generated. Describe any experiments used to assess the effect of a mutation and, where applicable, how potential secondary effects (e.g. second site T-DNA insertions, mosiacism, off-target gene editing) were examined.*
